# StrDiSeg: Adapter-Enhanced DINOv3 for Automated Ischemic Stroke Lesion Segmentation

**DOI:** 10.3390/bioengineering13020133

**Published:** 2026-01-23

**Authors:** Qiong Chen, Donghao Zhang, Yimin Chen, Siyuan Zhang, Yue Sun, Fabiano Reis, Li M. Li, Li Yuan, Huijuan Jin, Wu Qiu

**Affiliations:** 1 Ultrasound Diagnosis Department, Wuhan No. 1 Hospital, Wuhan 430000, China; qchen.wh@outlook.com; 2 School of Life Science and Technology, Huazhong University of Science and Technology, Wuhan 430074, China; zhangdonghao319@163.com (D.Z.); chenyiminch@gmail.com (Y.C.); wuqiu@hust.edu.cn (W.Q.); 3 Blood Optical Imaging Technology Center, Central Laboratory, Wuhan Blood Center, Wuhan 430030, China; siyuan.z@whblood.org.cn; 4 Faculty of Applied Sciences, Macao Polytechnic University, Macao 999078, China; yuesun@mpu.edu.mo; 5 Department of Radiology and Oncology, University of Campinas, Campinas 13083-888, Brazil; fabianoreis2@gmail.com; 6 Neuroimaging Laboratory, Department of Neurology, University of Campinas, Campinas 13083-887, Brazil; limin@unicamp.br; 7 Department of Ultrasound, Wuhan Children’s Hospital (Wuhan Maternal and Child Healthcare Hospital), Tongji Medical College, Huazhong University of Science and Technology, Wuhan 430014, China; 8 Department of Neurology, Union Hospital, Tongji Medical College, Huazhong University of Science and Technology, Wuhan 430030, China

**Keywords:** stroke segmentation, vision foundation model, finetuning

## Abstract

Deep vision foundation models such as DINOv3 offer strong visual representation capacity, but their direct deployment in medical image segmentation remains difficult due to the limited availability of annotated clinical data and the computational cost of full fine-tuning. This study proposes an adaptation framework called StrDiSeg that integrates lightweight bottleneck adapters between selected transformer layers of DINOv3, enabling task-specific learning while preserving pretrained knowledge. An attention-enhanced U-Net decoder with multi-scale feature fusion further refines the representations. Experiments were performed on two publicly available ischemic stroke lesion segmentation datasets—AISD (Non Contrast CT) and ISLES22 (DWI). The proposed method achieved Dice scores of 0.516 on AISD and 0.824 on ISLES22, outperforming baseline models and demonstrating strong robustness across different clinical imaging modalities. These results indicate that adapter-based fine-tuning provides a practical and computationally efficient strategy for leveraging large pretrained vision models in medical image segmentation.

## 1. Introduction

Acute ischemic stroke (AIS) is one of the leading causes of mortality and long-term disability worldwide, and rapid and accurate assessment of ischemic brain injury is essential for treatment selection and clinical prognosis. Early imaging evaluation plays a decisive role in determining treatment windows, thrombolysis eligibility, and predicting functional outcomes [1,2]. Non-contrast computed tomography (NCCT) and diffusion-weighted magnetic resonance imaging (DWI) are the most widely used modalities for ischemic lesion assessment [1]. Automatically and accurately segmenting stroke lesions from these images can greatly accelerate clinical workflows, reduce the variability associated with manual interpretation, and provide objective biomarkers for decision support [3,4].

However, ischemic lesions on NCCT and DWI imaging are highly heterogeneous in terms of appearance, location, size, and intensity distribution. Early lesions on NCCT frequently exhibit extremely subtle density changes, often approaching the limit of human perceptual discrimination, while DWI lesions may demonstrate sharp lesion boundaries but can vary significantly across patients [1]. Traditional medical image segmentation methods, such as level set, region growing, or active contour models, require hand-crafted image features and heuristic parameters that are sensitive to noise and image quality. These methods often struggle to generalize across different scanners, patient populations, or lesion subtypes, leading to inconsistent or unstable clinical performance [5,6,7].

Deep learning has transformed medical image analysis by enabling data-driven feature representation and end-to-end optimization [8]. Convolutional neural networks (CNNs), such as U-Net and its variants [9,10,11,12,13], have achieved significant advances in brain lesion segmentation by leveraging hierarchical feature extraction and skip-connection architectures to preserve spatial details. More recently, the emergence of vision transformer (ViT) models [14] has introduced a new paradigm for visual recognition, replacing local convolutional operators with global self-attention mechanisms capable of modeling large-scale context and long-range relationships. ViTs have demonstrated strong success when trained on extremely large image collections, inspiring a broad range of transformer-based models for medical segmentation tasks. Hybrid and hierarchical designs have further extended their representational power, leading to state-of-the-art results on many clinical imaging benchmarks [15,16,17].

Despite these advances, a major bottleneck remains: medical imaging datasets are typically orders of magnitude smaller than natural image datasets, causing strong domain gaps when directly transferring models trained on large-scale natural images. One conventional approach to address this challenge is to fine-tune only a small portion of the model, such as by introducing adapter modules or freezing most backbone layers to reduce the risk of overfitting [18,19]. While these lightweight transfer strategies are computationally efficient, they inherently assume that high-level visual concepts learned from natural images sufficiently align with medical visual structures. In ischemic stroke imaging, this assumption does not always hold. NCCT lesions are often low-contrast, highly subtle, and have completely different texture statistics compared with natural photographs [1]; therefore, shallow and mid-level features extracted from a frozen backbone may not provide sufficiently discriminative representations. As a result, limited fine-tuning may fail to adapt the pretrained model to the fundamental characteristics of stroke imaging.

With the growing availability of large-scale pretrained vision models, recent work has begun to explore fully unfreezing the backbone during adaptation to medical imaging tasks. Although this approach entails a substantial computational and parameter-optimization burden, it offers the major advantage of allowing every layer of the pretrained model to adjust to the new domain [20,21]. For tasks where visual appearance deviates drastically from natural photographs, such as ischemic lesion segmentation on NCCT or DWI, full fine-tuning may be necessary to recover optimal representational alignment. However, relatively few studies have systematically evaluated whether fully unfreezing large-scale transformer backbones indeed leads to improved performance in clinical disease segmentation, and how such large feature extractors should be efficiently integrated with task-specific decoders.

In this study, we address this question by investigating the use of a fully fine-tuned DINOv3 [22] Vision Transformer (ViT-B backbone distilled from the version of 7 billon parameters, approximately 85 milion parameters) for ischemic stroke lesion segmentation on two public datasets: AISD (NCCT) [23] and ISLES22 (DWI) [24]. Unlike parameter-efficient tuning approaches explored in many recent studies, we unfreeze the entire DINOv3 backbone and train all parameters end-to-end. Motivated by clinical observations and empirical findings, we hypothesize that ischemic lesion appearance differs strongly from natural image feature distributions, while fully unfreezing the model allows better adaptation of both low-level and high-level feature representations, and this adaptation will translate into significantly improved segmentation accuracy compared with partially frozen or only adapter-based approaches.

To decode the refined transformer features into pixel-accurate segmentation masks, we design a lightweight U-Net-style multi-scale decoder equipped with both spatial and channel attention modules to better integrate high-resolution local cues and deep semantic representations [25]. This decoder preserves fine structural details while mitigating the tendency of ViTs to lose spatial precision due to global tokenization. The full model is trained end-to-end using a hybrid loss combining Dice and binary cross-entropy objectives, balancing overlap alignment with voxelwise classification fidelity.

Our contributions are as follows:

1. We present a comprehensive investigation into end-to-end fine-tuning of a large pretrained vision transformer for ischemic stroke lesion segmentation on NCCT and MRI;
2. We design a multi-scale U-Net-style decoder with attention mechanisms for precise structural reconstruction from transformer features;
3. We demonstrate experimentally that full fine-tuning significantly improves segmentation performance over partial or lightweight transfer, suggesting that large pretrained on natural imagees visual backbones may require full representational adaptation in clinical imaging domains with strong modality shifts.

## 2. Materials and Methods

### 2.1. Datasets

This study evaluates the proposed segmentation framework on two public ischemic stroke datasets—AISD [23] and ISLES22 [24]. AISD contains Non-Contrast CT examinations collected from acute stroke patients within 24 h of symptom onset, whereas ISLES22 provides magnetic resonance imaging with expert delineations of stroke lesions in the acute to subacute phase. These two datasets allow comprehensive assessment of segmentation robustness across different acquisition environments and imaging modalities.

For AISD, we follow the official data split provided by the dataset authors. AISD contains 397 NCCT scans acquired from acute ischemic stroke patients whose imaging was performed within 24 h after symptom onset. All patients subsequently underwent diffusion-weighted MRI within the same 24-h window, which serves as the reference standard for lesion annotation. The CT scans were acquired with a slice thickness of 5 mm. Lesion masks were generated by a trained radiologist using MRI as a diagnostic reference, and subsequently double-reviewed and confirmed by a senior expert to ensure annotation reliability. Among the 397 cases, 345 scans are used for model development and validation, while the remaining 52 constitute the official held-out test set. The segmentation labels categorize tissues into five classes—remote, clearly visible acute infarct, blurred acute infarct, invisible acute lesion, and general infarction—where we follow common practice and consider all labels 1, 2, 3, 5 as positive infarct regions for supervised segmentation.

In contrast, the ISLES22 dataset provides a large, multi-center MRI cohort collected from multiple vendors and imaging platforms, reflecting the significant heterogeneity of real-world stroke imaging. This dataset includes 400 cases with substantial variability in lesion volume, distribution, number, and etiology. Patient MRI was acquired as part of clinical workflow for treatment planning, risk stratification, and outcome assessment. In this study, we use the 250 publicly available training subjects and randomly divide them into 200 training cases and 50 testing cases. Although full MRI studies provide diffusion-weighted imaging (DWI), apparent diffusion coefficient (ADC), and FLAIR sequences, we restrict model training to the DWI modality only.

### 2.2. Proposed Model Architecture

**Figure 1:**
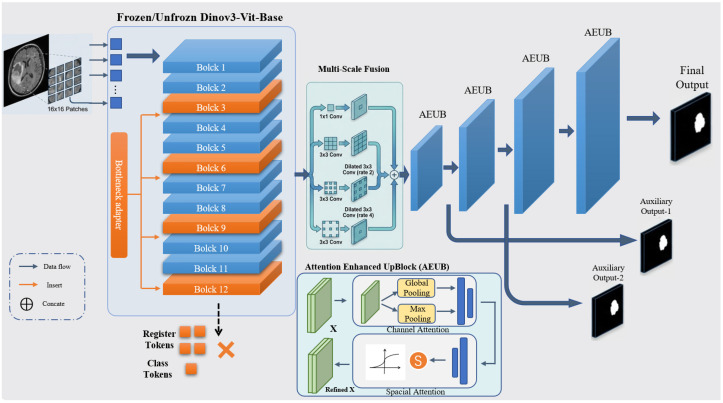
Overall architecture of the proposed framework. The framework features a hybrid 2D slice-based design consisting of three core modules: (Left) the pre-trained DINOv3 Vision Transformer (ViT-Base) encoder with 12 transformer blocks, where selected blocks are fine-tuned or integrated with bottleneck adapters for domain-specific feature extraction; (Right) an attention-enhanced U-Net-like decoder comprising four upsampling stages with multi-scale feature fusion and attention modulation to produce high-resolution segmentation masks. Blue arrows indicate the data flow between modules, while red arrows denote the insertion of bottleneck adapters into specific transformer blocks. Transformer blocks highlighted in red correspond to the adapted layers (Blocks 3, 6, 9, and 12). The symbol “×” denotes tokens that are disabled and not used in the feature extraction pipeline.

The proposed framework adapts the DINOv3 vision foundation model for ischemic stroke lesion segmentation. As shown in Figure 1, the architecture comprises three major components: (1) a pretrained DINOv3 encoder that extracts hierarchical visual representations; (2) lightweight bottleneck adapters inserted into selected Transformer layers to enable parameter-efficient domain adaptation; (3) an attention-enhanced U-Net decoder with multi-scale feature fusion for high-resolution lesion prediction. Since DINOv3 is pretrained on large-scale 2D natural images, the entire framework is designed as a slice-based 2D segmentation model, processing each axial NCCT or DWI slice independently. This design exploits the powerful pretrained representations of DINOv3 and supports efficient fine-tuning on limited medical datasets.

#### 2.2.1. Pretrained DINOv3 Encoder

DINOv3 [22] is a self-supervised vision foundation model trained on more than 1.4 billion natural images using a teacher–student distillation strategy. Unlike supervised models, DINOv3 learns robust visual representations by enforcing consistency between different views of an image, utilizing a centering and sharpening mechanism in the teacher network to avoid collapse. This self-supervised objective allows the model to capture high-level semantic features and precise spatial geometry without manual annotations. The distilled ViT-Base variant, containing approximately 85 million parameters, is employed as the backbone encoder in this study due to its strong representational capacity while low demand of memory.

Each 2D slice is partitioned into non-overlapping 16×16 patches that are flattened and linearly projected into 768-dimensional tokens. Learnable positional embeddings are added to retain spatial structure. Besides the patch tokens, the original architecture introduces global class and register tokens; however, only patch tokens are utilized for constructing spatial feature maps during decoding.The token sequence passes through 12 Transformer blocks, each consisting of multi-head self-attention, feed-forward networks, layer normalization, and residual connections. This hierarchical structure allows the encoder to capture both fine-grained local textures and long-range anatomical context, which are essential for detecting subtle ischemic abnormalities.

Two encoder training strategies are investigated: (1) Frozen Backbone: freezing all backbone parameters to preserve the generic semantic pretrained features, ensuring that the model relies entirely on the lightweight adapters for domain adaptation; and (2) Unfrozen Backbone: fine-tuning the backbone with a small learning rate ($1\times 10^{-5}$) to promote deep domain adaptation toward clinical imaging characteristics, allowing the large-scale pretrained weights to subtly align with the low-contrast gradients typical of ischemic stroke lesions in CT/MRI.

#### 2.2.2. Adapter-Based Fine-Tuning

**Figure 2:**
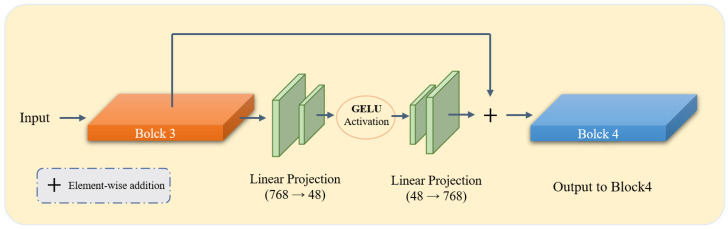
Detailed architecture of the proposed Bottleneck Adapter integrated within the DINOv3 Vision Transformer backbone. As shown, the adapters are strategically inserted at specific transformer blocks (3, 6, 9, and 12) to capture hierarchical features while maintaining the pre-trained knowledge. Each adapter consists of a down-projection layer, a nonlinear activation function (GELU), and an up-projection layer.

To enable parameter-efficient domain adaptation while preserving the pretrained representations, bottleneck adapters are inserted into the 3rd, 6th, 9th, and 12th Transformer layers. Each adapter adopts a lightweight residual bottleneck structure. Given a token feature vector $x\in R^{768}$, the adapter first projects it into a 48-dimensional latent space, applies GELU activation, and then restores it to the original dimension. The residual update is formulated as:

(1) y=x+B(x),

where B(·) denotes the bottleneck transformation consisting of a down-projection, GELU nonlinearity, and an up-projection. As illustrated in Figure 2, we employ the Gaussian Error Linear Unit (GELU) as the activation function within the bottleneck. Compared to the standard ReLU, GELU provides a smoother, non-monotonic, and differentiable alternative that weighs inputs by their percentile, which helps mitigate the vanishing gradient problem and enhances the stability of task-specific refinements during adaptation. This residual formulation ensures that the pretrained representation is preserved while allowing the model to learn task-specific refinements in a stable and controlled manner.

The adapters introduce only 298,176 additional parameters, corresponding to less than 0.35% of the encoder. Despite their lightweight design, they provide effective domain-specific modulation with minimal computational overhead. A hierarchical learning rate schedule is employed during training: $1\times 10^{-5}$ for the encoder backbone (if unfrozen), $1\times 10^{-4}$ for the adapters, and $1\times 10^{-4}$ for the decoder.

#### 2.2.3. Attention-Enhanced U-Net Decoder

Following the encoder, the patch tokens are reshaped into a spatial feature map of size H/16×W/16. A multi-scale feature aggregation module is first applied, comprising parallel convolutional branches with receptive fields of 1×1, 3×3, and dilated 3×3 kernels with dilation rates of 2 and 4. Each branch reduces feature dimensionality to 192 channels, and their concatenated output is refined using a 3×3 convolution with a residual connection.

The decoder utilizes a hierarchical upsampling structure where each stage incorporates a serial dual-path attention mechanism to facilitate Attention-based Feature Decoupling and Detection (AFDD) [26]. This design ensures that the foundation model’s features are refined to isolate subtle lesion signals from complex anatomical backgrounds. Specifically, after the transpose convolution $f_{up}$, the feature map $F\in R^{C\times H\times W}$ is sequentially recalibrated through channel-wise and spatial-wise dimensions [25].

First, the channel attention component recalibrates inter-channel dependencies to prioritize semantic features indicative of ischemia. By aggregating global spatial information via both average and maximum pooling, the channel weight map $M_{c}(F)$ is formulated using a shared multi-layer network:

(2) $$M_{c}(F)=\sigma \left(MLP\left(AvgPool\left(F\right)\right)+MLP\left(MaxPool\left(F\right)\right)\right)$$

The intermediate feature is then generated as $F_{c}=M_{c}(F)⊗F$. To further suppress non-pathological noise, a spatial contextual refinement module is applied. It captures the local distribution of the infarct by concatenating channel-wise statistics and applying a convolution $f_{spatial}$ with a robust 7×7 receptive field:

(3) $$M_{s}(F_{c})=\sigma \left(f_{spatial}^{7\times 7}\left(\left[AvgPool\left(F_{c}\right);MaxPool\left(F_{c}\right)\right]\right)\right)$$

The final refined output $F_{out}=M_{s}(F_{c})⊗F_{c}$ provides the necessary inductive bias for delineating fragmented or low-contrast lesions. In our clinical context, this sequential refinement is important: the channel-wise path identifies “what” pathological intensity profiles are present, while the spatial path constrains the model’s focus to “where” the potential infarct zone lies. This explicit decoupling strategy aligns with recent advances in attention-guided medical feature refinement [13,27], effectively bridging the domain gap between natural image priors and clinical stroke morphology.

To improve optimization stability, auxiliary segmentation heads are attached to the first two upsampling stages, providing deep supervision at 1/8 and 1/4 resolutions. The final prediction is generated using a 3×3 convolution followed by a 1×1 classifier and upsampled via bilinear interpolation to the input size, yielding the final voxel-wise lesion probability map.

### 2.3. Loss Function

The network is trained using a hybrid loss that addresses both regional overlap and pixel-wise accuracy. The primary loss combines Dice loss and binary cross-entropy (BCE). Dice loss mitigates class imbalance, emphasizing correct overlap of predicted and ground truth lesions, while BCE penalizes misclassified pixels. Deep supervision is applied to decoder intermediate outputs, weighted at 0.3 relative to the main loss, yielding the total loss:

(4) $$L_{total}=L_{Dice}+L_{BCE}+0.3\sum_{i}L_{aux,i},$$

where $L_{aux,i}$ denotes losses at intermediate decoder stages. The auxiliary-loss weight of 0.3 is chosen to encourage stable gradient propagation from intermediate layers without overwhelming the main segmentation objective, providing regularization while keeping the optimization primarily guided by the final prediction [11,28].

### 2.4. Evaluation Metrics and Implementation Details

#### 2.4.1. Evaluation Metrics

To provide a rigorous quantitative assessment, we formally define the metrics used to evaluate model performance across 2D slice-level and 3D volume-level dimensions. Let *P* and *G* represent the set of foreground pixels in the predicted segmentation mask and the ground truth, respectively. The Dice Similarity Coefficient (DSC) and Intersection over Union (IoU) are defined as:

(5) $$DSC\left(P,G\right)=\frac{2|P\cap G|}{\left|P|+|G\right|},\;IoU\left(P,G\right)=\frac{\left|P\cap G\right|}{\left|P\cup G\right|}$$

Boundary accuracy was assessed using the Hausdorff Distance (HD) and its 95th percentile variant (HD95). Let $d(p,G)=\min_{g\in G}||\;p-{g\;||}_{2}$ denote the shortest Euclidean distance from a point *p* to the set *G*. The HD95 is defined as the 95th percentile ($P_{95}$) of the symmetric distances to suppress the influence of segmentation outliers:

(6) $$HD95(P,G)=\text{max}\left(P95p\in Pd(p,G),P95g\in Gd(g,P)\right)$$

For 3D volumetric consistency, we employed the Volumetric Difference Percentage (VDP) and Volumetric Correlation (VC). Based on our implementation, VDP measures the proportion of the ground-truth lesion volume that is not correctly covered by the prediction:

(7) $$VDP=\frac{\left|G|-|P\cap G\right|}{\left|G\right|}\times 100\%$$

To evaluate the linear consistency of lesion across the dataset, VC is defined as the Pearson correlation coefficient between the predicted volume and the ground truth:

(8) $$VC=\frac{\sum \left(V_{P}-{\bar{V}}_{P}\right)\left(V_{G}-{\bar{V}}_{G}\right)}{\sqrt{\sum {\left(V_{P}-{\bar{V}}_{P}\right)}^{2}\sum {\left(V_{G}-{\bar{V}}_{G}\right)}^{2}}}$$

where $V_{P}$ and $V_{G}$ denote the total counts of positive pixels, and $\bar{V}$ represents their mean values. Together, these metrics provide a comprehensive evaluation of both spatial precision and clinical volumetric reliability.

#### 2.4.2. Implementation Details

All baseline models (SegResNet, UNETR, SwinUNETR, nnU-Net, Unet++, and AttnUnet) were trained from scratch or using their official pre-trained weights under identical experimental conditions. All input slices were resized to 224×224 pixels to align with the input resolution of DINOv3. All experiments were implemented in PyTorch 2.7.0 and executed on an NVIDIA RTX 4090 GPU (Nvidia, Santa Clara, CA, USA) with a fixed batch size of 8.

The data augmentation policy was standardized across all models to account for stroke lesion, including: (1) Random rotations within $\pm 10^{\circ }$; (2) Horizontal flipping (probability = 0.5); and (3) Intensity scaling and Gaussian noise (σ=0.01) to simulate varying clinical image qualities. For the training schedule, all models were trained for 100 epochs, which was sufficient for convergence across all architectures. We utilized the Adam optimizer ($\beta_{1}=0.9,\beta_{2}=0.999$) with a weight decay of $1\times 10^{-5}$. For the baselines, the learning rate was optimized via a grid search within $\left[1\times 10^{-3},1\times 10^{-5}\right]$ to ensure they reached their peak performance. For our proposed model, the initial learning rate was $1\times 10^{-4}$ for the adapters and $1\times 10^{-5}$ for the backbone fine-tuning, both controlled by a ReduceLROnPlateau scheduler (factor 0.5, patience 10).

The data augmentation policy was specifically tailored to the clinical characteristics of stroke lesions in NCCT and DWI scans. Random rotations were restricted to $\pm 10^{\circ }$ because head tilt in clinical scanners is typically minor; excessive rotation would create anatomically unrealistic orientations. Horizontal flipping was utilized with a probability of 0.5 to leverage the bilateral symmetry of the brain, encouraging the model to learn hemispheric-independent lesion features. For intensity-based transforms, Gaussian noise (σ=0.01) and Intensity scaling were applied to simulate the inter-scanner variability and noise levels common in emergency clinical settings. These conservative parameter ranges ensure that the augmented samples remain within the manifold of plausible clinical presentations while improving the model’s generalization to subtle intensity variations.

## 3. Results

### 3.1. Quantitative Performance

Table 1 and Table 2 summarize the quantitative results on the AISD (NCCT) and ISLES22 (DWI) datasets, respectively. On AISD, our method achieves a Dice score of 0.516, outperforming representative CNN-based methods (U-Net, UNet++, SegResNet) and Transformer-based models (UNETR, SwinUNETR). The gains are particularly pronounced on NCCT images, where low contrast and heterogeneous intensity distributions pose challenges for conventional architectures. Our approach also yields the best HD95 and IoU among all comparisons, indicating improved overlap and boundary localization.

For the ISLES22 DWI dataset, our method attains a Dice score of 0.824 and better IoU, VC, and VDP, demonstrating both accurate lesion contour prediction and highly consistent lesion volume estimation.

To evaluate the impact of different training strategies, we compared two specific regimes: the “Backbone Frozen” (Adapter-only) approach, where the DINOv3 encoder is used as a fixed feature extractor while only training the adapters and decoder, and the entire network is fine-tuned to adapt to the medical domain. As summarized in Table 1 and Table 2, fine-tuning the entire network outperforms the adapter-only variant across all metrics. For instance, on the AISD dataset, the Dice score increases from 0.372 to 0.516. This substantial gain confirms that while DINOv3 provides a robust parameter initialization, the inherent domain gap between natural scenes and clinical NCCT/DWI scans necessitates unfreezing the backbone to effectively refine representations for subtle lesion morphology.

### 3.2. Qualitative Visualization

**Figure 3:**
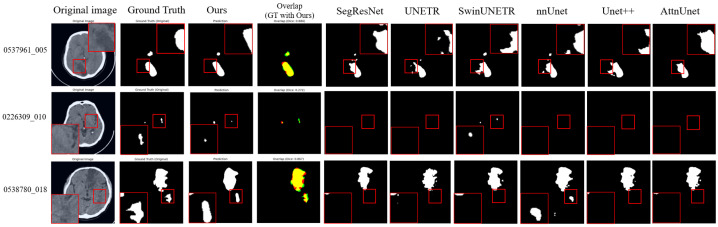
Visualization results on the AISD dataset. In the overlap column, red indicates our model predictions, green represents the ground truth, and yellow shows their overlapping regions. The magnified regions (red boxes) provide a zoom-in view of the segmented lesions, highlighting our model’s ability to accurately delineate blurred boundaries and detect small infarcts in challenging low-contrast imaging conditions.

**Figure 4:**
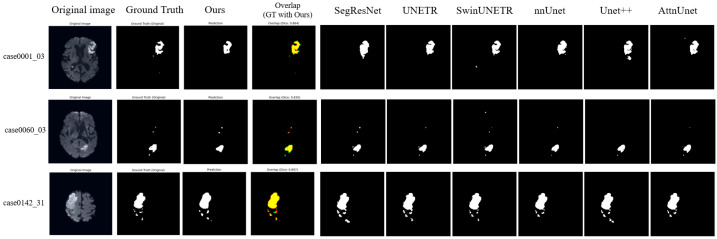
Visualization Results on ISLES22 dataset. In the overlap column, red indicates our model predictions, green represents the ground truth, and yellow shows their overlapping regions. While most models perform well on high-contrast DWI sequences, our method demonstrates better volumetric consistency and reduced false positives in the peripheral regions of the infarct, as evidenced by the high overlap with the ground truth.

Representative segmentation results (Figure 3 and Figure 4) demonstrate that the proposed method accurately delineates ischemic lesions, providing sharper boundaries and more precise identification of small or fragmented infarcts compared to baseline architectures. The integration of multi-scale attention and deep supervision effectively suppresses false positives in healthy tissue and preserves spatial consistency across slices. These visual improvements are particularly evident in low-contrast NCCT images, highlighting the model’s capability to robustly capture lesion morphology under challenging imaging conditions.

**Figure 5:**
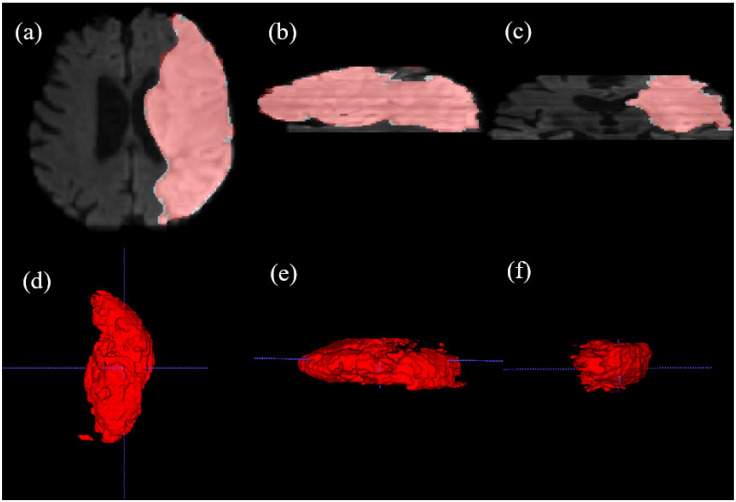
Multi-planar visualization and multi-angle 3D volumetric reconstruction of a stroke lesion from the ISLES22 dataset, generated using ITK-SNAP v3.6.0. (**a**) Axial plane (AP-RL), (**b**) Sagittal plane (SI-AP), and (**c**) Coronal plane (SI-RL) views, where red contours indicate our model’s predictions. The results demonstrate the spatial consistency of our slice-based approach. (**d**–**f**) 3D volumetric renderings of the segmented lesion viewed from different orientations, illustrating the model’s ability to reconstruct the complex 3D morphology of the infarct. Blue dashed lines indicate standard reference lines. This comprehensive visualization confirms that the 2D DINOv3-based features maintain robust 3D clinical consistency.

To further evaluate the spatial continuity of our 2D slice-based segmentation, a 3D reconstruction of a stroke lesion from the ISLES22 dataset was performed (Figure 5). The resulting 3D volume demonstrates that our model maintains morphological consistency across contiguous axial slices, capturing the volumetric extent of the infarct.

**Figure 6:**
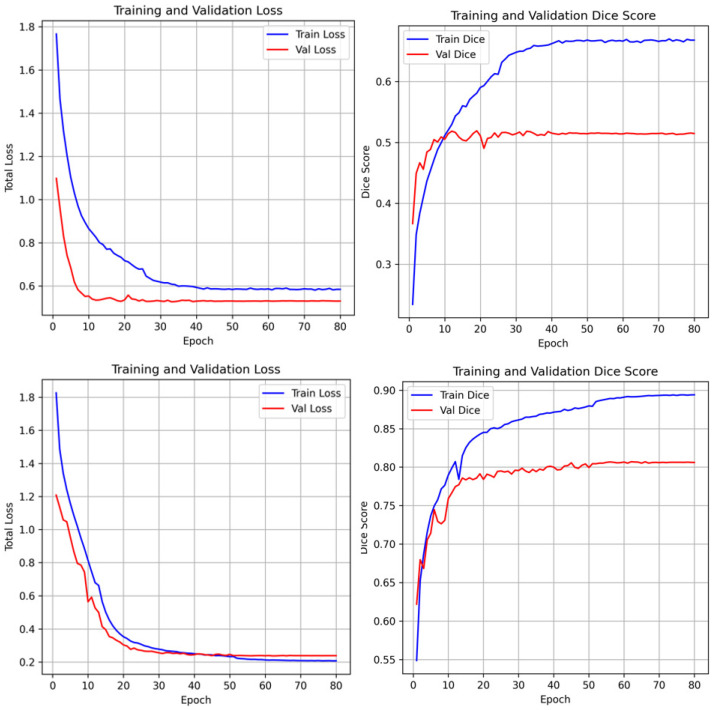
Training and validation loss curves and dice scores of the unfrozen model. The stable convergence and stable increase of dice scores across epochs demonstrates that the foundation model adaptation avoids overfitting despite the specialized nature of the clinical datasets.

**Figure 7:**
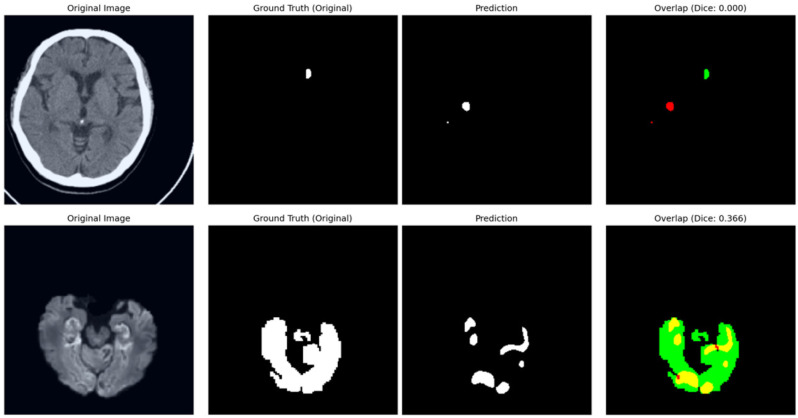
Analysis of typical failure cases. In the overlap column, red contours denote model predictions, green areas represent the ground truth, and yellow indicates their intersection. (**Top**) In the AISD dataset, the model occasionally fails to localise ultra-small, “point-like” lesions due to low signal-to-noise ratios in NCCT. (**Bottom**) In the ISLES22 dataset, performance can be affected by the intensity similarity between infarct tissues and healthy structures, leading to under-segmentation.

The training dynamics of the proposed framework are illustrated in Figure 6. Both training and validation losses exhibit a consistent downward trend, signifying stable convergence and robust optimization. To provide a transparent evaluation of the model’s limitations, typical failure cases are presented in Figure 7. In the NCCT-based AISD dataset, the model occasionally misses extremely small infarcts where the intensity profile is nearly indistinguishable from background noise. In the DWI-based ISLES22 dataset, challenges not only from small lesion but also arise from high-intensity artifacts or anatomical structures with appearances similar to acute lesions, leading to localized boundary confusion. These limitations stem from the inherent resolution constraints of 2D imaging and signal heterogeneity, rather than architectural deficiencies. Future work incorporating multi-modal fusion or 3D contextual refinement may further mitigate these challenges.

### 3.3. Ablation Experiments

To assess the contribution of individual components, ablation studies were conducted on the AISD dataset and the results are summarized in Table 3. The Full model achieves the highest Dice score of 0.5161, which validates the synergistic effect of the proposed architectural components. First, comparing Setting 3 with the Full model, removing the adapter modules from the Transformer backbone led to a reduction in Dice score, highlighting their critical role in domain-specific feature adaptation without disrupting the pretrained foundational knowledge. Second, as shown in Setting 4, excluding the attention mechanisms in the decoder resulted in reduced boundary discrimination and increased misclassification in background tissue, demonstrating the importance of channel-wise and spatial-wise attention for filtering noise and focusing on fine-grained lesion segmentation. Finally, Setting 2 demonstrates that freezing the backbone during training greatly degraded performance (decreasing from 0.5161 to 0.3719), indicating that while the DINOv3 encoder provides powerful generic features, its natural-image priors require limited fine-tuning to further enhance feature alignment with the specific intensity distributions of medical imaging characteristics.

Furthermore, we investigated the impact of the loss function configuration and the auxiliary supervision weight, as detailed in Table 4. Comparing Setting 3 with Settings 1 and 2, the combination of Dice and Binary Cross-Entropy (BCE) loss yields a higher Dice score (0.5119) than using either term alone, confirming that Dice loss addresses class imbalance while BCE ensures pixel-level BCE convergence. To justify the auxiliary supervision, Setting 4 (Full model configuration) incorporates deep supervision with a weighting factor $\lambda_{aux}=0.3$, resulting in the optimal score of 0.5161. A parameter study comparing $\lambda_{aux}=0.3$ (Setting 4) and $\lambda_{aux}=0.8$ (Setting 5) shows that while auxiliary supervision is essential for stabilizing early layer training, an excessively high weight can slightly detract from the final performance. The results demonstrate that $\lambda_{aux}=0.3$ provides the relatively better balance between deep feature guidance and final segmentation accuracy.

The ablation experiments confirm that the combination of pretrained foundation features, lightweight adapters, and attention-guided multi-scale decoding synergistically contributes to the improved segmentation accuracy and robustness of the proposed framework.The consistent performance drop observed across all ablation settings (Settings 2, 3, and 4) further justifies the necessity of each integrated module.

The observed performance gains are more prominent in the CT-based AISD dataset, reflecting the model’s ability to overcome the inherent challenges posed by CT imaging, including low contrast and heterogeneous appearance of ischemic lesions. In contrast, the high baseline performance on ISLES22 suggests that DWI provides inherently higher lesion contrast, making task adaptation less demanding but still benefiting from the proposed adapter-based fine-tuning strategy. These trends underscore the versatility of foundation model adaptation across diverse imaging modalities and lesion characteristics.

## 4. Discussion

The results of this study demonstrate that the adaptation of vision foundation models is an effective strategy for ischemic stroke lesion segmentation. By introducing lightweight bottleneck adapters into DINOv3, pretrained representations learned from large-scale natural images can be successfully transferred to clinical NCCT and DWI scans without requiring extensive medical datasets. The attention-enhanced U-Net decoder further improves lesion localization by refining feature discriminability through channel-wise and spatial attention mechanisms. Compared with conventional CNN or Transformer-based medical segmentation models, the proposed StrDiSeg framwork shows better generalization across heterogeneous lesion distributions, indicating that large-scale pretrained models encode transferable visual priors relevant to clinical imaging.

Furthermore, the clinical practicality of our StrDiSeg framework is underpinned by the choice of the distilled ViT-Base variant. While recently proposed models like the Encoder-only Mask Transformer (EoMT) [29] suggest that architectural simplicity can lead to higher speeds, such gains are often most prominent at scales (e.g., ViT-Large/Huge) and require massive pre-training datasets to internalize segmentation biases. Similarly, while Masked Autoencoders (MAE) [30] share a comparable ViT-Base architecture, their reconstruction-based pre-training often lacks the robust semantic discriminability of DINOv3. In contrast, our approach utilizes a distilled ViT-Base (85M parameters), which is significantly more lightweight than frontier models like InternVL [31,32,33]. By inheriting high-quality semantic priors from DINOv3, the model achieves a balance between parameter efficiency and diagnostic precision. This efficiency translates into rapid clinical inference, with an average processing time of 2.88 s per patient sequence on an RTX 4090 GPU.

Regarding the architecture, we leverage the unfrozen DINOv3 backbone as a highly specialized feature extractor. Unlike strategies that force a plain encoder to learn complex segmentation biases from scratch—as seen in EoMT—our method benefits from the extensive “visual feature cultivation” performed during DINOv3’s natural image pre-training. By using these pretrained weights as a better initialization for the fine-tuning stage, the encoder possesses an innate ability to capture fine-grained semantic structures even before seeing medical data. Our strategy, which incorporates lightweight bottleneck adapters (<0.35% parameters) and an unfreezing schedule, allows the model to refine these rich priors for the stroke domain. This approach ensures that the necessary inductive bias for lesion delineation is maintained even with limited labeled data, effectively mitigating the overfitting risks typically associated with training large-scale transformers on small medical cohorts.

Despite these promising results, several limitations remain. First, the current approach processes each slice independently, without leveraging full 3D spatial continuity. Second, while AISD and ISLES22 cover major imaging modalities in stroke, broader multi-center validation is needed to assess robustness across diverse clinical settings. Finally, the integration of clinical metadata, such as NIHSS scores or stroke onset time, could further enhance predictive power and interpretability in future studies.

## 5. Conclusions

In this work, we present StrDiSeg, a fine-tuning framework for adapting the DINOv3 vision foundation model to ischemic stroke lesion segmentation on NCCT and DWI. By incorporating bottleneck adapters and an attention-enhanced decoder, the proposed framework achieves competitive performance on AISD and ISLES22 datasets while maintaining a low level of complexity. The study highlights the potential of large-scale pretrained foundation models to provide transferable visual representations for clinical neuroimaging tasks. Our approach establishes a simple yet effective baseline for future medical AI systems that leverage self-supervised vision foundation models, offering a practical pathway for scaling robust lesion segmentation without extensive annotated datasets.

## Figures and Tables

**Table 1:** Quantitative Results of Brain NCCT Dataset AISD.

Model	Dice ↑	HD/HD95 ↓	IoU ↑	VC ↑	VDP ↓
SegResNet [12]	0.423	3.807/3.717	0.337	0.574	0.576
UNETR [15]	0.347	4.011/3.986	0.261	0.410	0.673
SwinUNETR [17]	0.447	3.744/3.687	0.351	0.541	0.565
nnUNet [10]	0.453	3.770/3.691	0.355	0.535	0.547
UNet++ [11]	0.441	3.759/3.677	0.352	0.585	0.540
AttnUnet [13]	0.428	3.795/3.687	0.349	0.608	0.563
Ours(Backbone Frozen)	0.372	4.080/4.077	0.269	0.383	0.552
Ours	0.516	3.493/3.432	0.400	0.528	0.429

Notes: Arrows indicate the desired trend: ↑ higher values are better, ↓ lower values are better. Underline highlights the best-performing results among all models. Dice: Dice similarity coefficient; HD/HD95: Hausdorff Distance / 95th percentile; IoU: Intersection over Union; VC: Volume Correlation; VDP: Volume Difference Percentage.

**Table 2:** Quantitative Results of Brain DWI Dataset ISLES 22’.

Model	Dice ↑	HD/HD95 ↓	IoU ↑	VC ↑	VDP ↓
SegResNet [12]	0.805	3.018/2.445	0.695	0.810	0.175
UNETR [15]	0.791	3.197/2.660	0.678	0.796	0.190
SwinUNETR [17]	0.800	3.059/2.525	0.688	0.804	0.204
nnUNet [10]	0.818	2.960/2.316	0.712	0.822	0.167
UNet++ [11]	0.814	2.985/2.354	0.706	0.817	0.178
AttnUnet [13]	0.813	3.018/2.446	0.704	0.817	0.171
Ours(Backbone Frozen)	0.741	3.342/3.067	0.610	0.743	0.229
Ours	0.824	3.096/2.390	0.716	0.826	0.155

Notes: ↑ indicates higher values are better, ↓ indicates lower values are better. Underline highlights the best-performing results among all models. Dice: Dice similarity coefficient; HD/HD95: Hausdorff Distance / 95th percentile; IoU: Intersection over Union; VC: Volume Correlation; VDP: Volume Difference Percentage.

**Table 3:** Results of components ablation study on AISD.

Setting	Backbone Trainable	Adapter	Decoder Attention	Dice ↑
(1) All closed	×	×	×	0.3405
(2) Frozen backbone	×	✓	✓	0.3719
(3) No adapter	✓	×	✓	0.5158
(4) No decoder attention	✓	✓	×	0.5135
**Full model**	✓	✓	✓	0.5161

Notes: ↑ indicate higher values are better. Underline highlights the best-performing results among all settings. The symbol × denotes the absence of a component, while ✓ indicates its presence.

**Table 4:** Results of loss function ablation study on AISD.

Setting	Dice	BCE	Auxiliary	Dice ↑
(1) Only Dice	✓	×	×	0.5088
(2) Only BCE	×	✓	×	0.4429
(3) Dice + BCE	✓	✓	×	0.5119
(4) **Dice + BCE + 0.3aux**	✓	✓	✓	**0.5161 **
(5) Dice + BCE + 0.8aux	✓	✓	✓	0.5159

Notes: ↑ indicates that higher values are better. Underline highlights the best-performing results among all settings. The symbol × denotes the absence of a component, while ✓ indicates its presence. Boldface highlights Setting 4, which corresponds to our final model (the Full model described in Table 3).

## Data Availability

The research data generated or analyzed during the study are available from the corresponding author upon reasonable request.

## Abbreviations

The following abbreviations are used in this manuscript:

NCCT	Non-contrast computed tomography
DWI	Diffusion-weighted imaging
MRI	Magnetic resonance imaging
ViT-B	Vision transformer—base
CNNs	Convolutional neural networks
AIS	Acute ischemic stroke
